# A Body Composition Aging Clock And Its Multiomic Profiles

**DOI:** 10.1093/geroni/igaf122.4402

**Published:** 2025-12-31

**Authors:** Liming Zhang, Xueqing Jia, Maoyao Xia, Shuli Jia, Jesse Poganik, Xia Jiang, Meiling Ge, Zuyun Liu

**Affiliations:** Zhejiang University, Hangzhou, Zhejiang, China (People’s Republic); Zhejiang university, Hangzhou, Zhejiang, China (People’s Republic); Sichuan University, Chengdu, Sichuan, China (People’s Republic); Sichuan University, Chengdu, Zhejiang, China (People’s Republic); Harvard Medical School, Boston, Massachusetts, United States; Sichuan University, Chengdu, Sichuan, China (People’s Republic); Sichuan University, Chengdu, Sichuan, China (People’s Republic); Zhejiang University, Hangzhou, Zhejiang, China (People’s Republic)

## Abstract

Body composition represent a fundamental and physiologically relevant dimension of aging. Leveraging deep body composition phenotypic data from a Chinese cohort, we developed BodycomBA, a novel aging clock, and BodycomBAA for assessing aging acceleration. BodycomBA showed robust correlations with chronological age (CA), and BodycomBAA exhibited strong efficacy in capturing a wide spectrum of physical health conditions. These findings were validated in two large independent cohorts (n = 9,256 and n = 3,403). Additionally, BodycomBAA was effective in discriminating early-onset cases of age-related diseases, and incorporating BodycomBA into models of conventional risk factors (e.g., CA, smoking, and drinking) enhanced the diagnostic power for these diseases. By integrating multiomic data, we identified 350 DNAm sites, 68 proteins, 41 metabolites, and 257 gut microbiota significantly associated with BodycomBAA and elucidated their related biological pathways. Calculating the similarity network between these pathways further identified 2 connected (integrin binding-focal adhesion pathways act as bridges between the 2 modules) and 8 independent functional modules for proteome and DNA methylome, respectively. Subsequent cross-omics analysis revealed interconnected networks of molecular pairs and core molecules (e.g., PLOD1 protein) underlying BodycomBAA. Interestingly, the omic-inferred BodycomBA models demonstrated enhanced capability in elucidating the heterogeneity of physical health conditions (e.g., functions of liver, kidney, and metabolism) compared to the actual BodycomBA. Collectively, this study presents a highly adaptable and valid aging clock derived from body composition data. Its multiomic profiles provide insights into aging biology and a valuable framework for precisely stratifying individuals at risk of accelerated aging, paving the way for personalized anti-aging interventions.

